# EVALUATION OF ETHICAL IN INSTRUCTIONS TO AUTHORS OF BRAZILIAN SURGICAL JOURNALS

**DOI:** 10.1590/S0102-6720201500040007

**Published:** 2015

**Authors:** Renan Kleber Costa TEIXEIRA, Vitor Nagai YAMAKI, Ruy Victor Simões PONTES, Marcus Vinicius Henriques BRITO, José Antonio Cordero da SILVA

**Affiliations:** Experimental Surgery Laboratory, Faculty of Medicine, State University of Pará, Belém, PA, Brazil.

**Keywords:** Bioethics, Journal article, Surgery

## Abstract

**Background:**

The instructions to authors are the only means of communication between researchers and the editorial standards of a scientific journal. One of the mandatory items to be contained therein is about the ethical part, to prevent new research to carry out abuses with the enrolled on the research are published and stimulated.

**Aim:**

To verify the ethical questions on the guidelines of Brazilian surgical journals

**Method:**

Thirteen selected journals were divided into two groups: general surgery (n=3), and surgical specialty (n=10). The instructions to authors were analyzed by the quote of ethical requirements based on a specific research protocol, ranging from zero to six points.

**Results:**

The average score of the general surgery group was similar than that of the surgical specialty group (3.66±0.57 *vs* 3.30±1.15, p=0.6154). When each ethical requirement was compared between the groups, there was no significant difference between the ethical requirements (p<0.05).

**Conclusion:**

There was respect for most ethical questions evaluated, with no difference between the journals of general or specialty surgery.

## Abstract (pt)

**Racional:**

As instruções aos autores são a principal comunicação entre os pesquisadores e as políticas editoriais. Um dos itens obrigatórios que devem conter é sobre a parte ética, para evitar que novas pesquisas que realizem abusos com os incluídos na pesquisa sejam publicadas e estimuladas.

**Objetivo:**

Verificar os quesitos éticos das instruções aos autores de revistas cirúrgicas nacionais.

**Método:**

Treze revistas escolhidas aleatoriamente do campo cirúrgico foram selecionadas. As instruções aos autores foram analisadas com base em protocolo de pesquisa contendo seis itens que devem obrigatoriamente conter nas instruções aos autores.

**Resultados:**

A média da pontuação do grupo de revistas gerais foi semelhante que o grupo de revistas sobre especialidades cirúrgicas (3,66±0,57*vs* 3,30±1,15, p=0,6154). Quando cada quesito ético foi comparado entre os grupos, não houve diferença significativa entre os quesitos estudados (p>0,05).

**Conclusão:**

Houve respeito pela maioria dos quesitos éticos avaliados, sem diferença entre as revistas de cunho geral ou especializadas.

## INTRODUCTION

Ethical principles have strongly changed in biomedical researchers overtime. Historically, the desire to clarify unanswered questions in the past was overvalued against welfare of participants. For example, pregnants were exposed to radiation to assess effects in fetuses^4^; the Tuskegee^1^ episode, experiments were carried out with poor and black people to observe the natural history of syphilis; even though, penicilin had already been defined as reference treatment. 

First international effort against those ethical misconducts was the Nuremberg code^7^. There were proposed ten guidelines that should be respected in research designs. However, those guidelines were not enough to minimize abuses against human beings. In 1964, the Helsinki^5^ declaration suggested the ethical review board that every research should be submitted in advance for evaluation and approval. In addition, review board approval declaration should be required for publishing a paper.

Based on those international recommendations, Brazilian regulations about researches were compiled in the 196/96 resolution from the Ministry of Health^3^^,^. Research Ethics Committees were created to approve and oversee ethical aspects in projects involving participants. Recently, the 196/96 resolution was switched by the 466/12^8^ with several updates and improvements about ethics in research. 

In process of preparing and formatting a paper to be published, the Instructions for Authors section is the only media of communication between authors and editorial board. Therefore, ethical requisites should be clearly informed in journals guidelines^14^. Nevertheless, papers^6^^,^ 9 ^-^ 11 ^,^ 13 ^,^ 14  have shown that critical ethical aspects, such as IRB approval, have not been requested in the Instructions for Authors.

This research is the first one addressing ethics the surgery field. Given that recent update in the Brazilian resolutions and the importance of ethical regulations in researches, were assessed instructions provided in Brazilian journals to examine their ethical profile.

## METHODS

This study was cross-sectional and observational. The sample consisted of 13 Brazilian medical journals distributed in two groups pursuant general surgery (Acta Cirúrgica Brasileira, Arquivos Brasileiros de Cirurgia Digestiva e Revista do Colégio Brasileiro de Cirurgiões) and surgical specialty (Acta Ortopédica Brasileira, Arquivos Brasileiros de Oftalmologia, Coluna/Columna, International Archives of Otorhinolaryngology, International Brazilian Journal of Urology, Jornal Vascular Brasileiro, Journal of Coloproctology, Revista Brasileira de Cirurgia Cardiovascular, Revista Brasileira de Oftalmologia e Revista Brasileira de Ortopedia).

The selection of the national surgical journals was performed at the site of Scientific Electronic Library Online (Scielo), where were selected all journals on the Brazilian collection bases, according to area "Health Sciences". After, were selected only the journals on surgical nature.

A score system was used in order to evaluate the ethical requirements in the instructions to the authors of the selected journals. The protocol used was an adaptation to the current legislation of the protocol proposed by Teixeira et al.^14^ In that protocol, there are six ethical requirements that should be described in the instructions to the authors as follows: 1) approval by the Research Ethics Committee; 2) reference to the fact that the research follows the precepts of the Declaration of Helsinki; 3) reference to the fact that the research is in compliance with the Resolution 466/12 of the National Health Council of the Brazilian Ministry of Health; 4) authorization from the subject agreeing to participate in the research through the informed consent; 5) information about the authors' conflicts of interest; and 6) request for registration of clinical trials in the Brazilian Clinical Trials Registry. One point was attributed to each ethical requirement identified in the instructions to authors; thus, a journal's score may vary from zero to six points. Was checked how many journals already cite the resolution 196/96.

In order to perform the statistical analysis, Student's t-test was used to compare the scores of several journals from the general and specialty surgery, and Fisher's exact test was used to compare each requirement individually between the groups. A p-value < 0.05 was adopted for significance.

## RESULTS

The average score of journals of the general surgery group was 3.66±0.57; the lowest was 3, and the highest, 4. The average score of the surgical specialty group was 3.30 ±1.15; the lowest was 1, and the highest, 5. There was no statistically significant difference between the groups (p=0.6154). The distribution of the scores between both study groups is described in Table 1.

For the studied requirements (Table 2), none proved to be statistically significant between the groups. In the first studied requirement, regarding the research assessment by a Research Ethics Committee, all journals of general surgery group had this requirement to the authors in their instructions, while eight (80%) journals of surgical specialty group required this approval. 

**TABLE 1:** - Distribution of the number of journals of the groups in the differents scores

**Note/Group**	**General surgery**	**Surgical specialty**
Zero	0 (0%)	0 (0%)
One	0 (0%)	1 (10%)
Two	0 (0%)	1 (10%)
Three	1 (33,34%)	3 (30%)
Four	2 (66,66%)	4 (40%)
Five	0 (0%)	1 (10%)
Six	0 (0%)	0 (0%)
Average	3,66	3,30

p=0.61 (t de Student)

**TABELA 2:** - Comparison between the presence and absence of each requirement in the groups

**Question/Group**	**General surgery**	**Surgical specialty**
Approval by a committee	3 (100%)	8 (80%)
Declaration of Helsinki	1 (34%)	5 (50%)
Resolution 466/12	1 (34%)	0 (0%)
Use of an informed consent	1 (34%)	2 (20%)
Disclosure of conflicts of interest	3 (100%)	10 (100%)
Brazilian clinical trials registry	2 (66%)	8 (80%)

p>0,05 (Fischer's exact test)

Regarding the requirement to mention the declaration of Helsinki and resolution 466/12 of the National Health Council of the Ministry of Health, in general surgical group, only one requested both; already in specialty group, a half requested the declaration of Helsinki and none met the resolution 466/12. Five journals (38%) cited resolution 196/96 in their guidelines. Regarding the requirement that interviewees should sign an informed consent, one (34%) journal of the general surgery group had this requirement, while in the surgical specialty group two (20%). 

All journals, regardless of group, required to announce the conflicts of interest of researchers and the researchers' conflicts of interest should be disclosed. With respect to the registration of clinical trials with the Brazilian Clinical Trials Registry, two journals (67%) in the general group and eight (80%) in the specialty group required such registration. 

## DISCUSSION

In most cases, the instructions to authors are the only means of communication between researchers and the editorial standards of a scientific journal^13^. Such document should inform a potential author regarding everything they should know before submitting an article for publication. The inclusion of ethical prerogatives in this space is essential not only to avoid ethical and bioethical atrocities but also to ensure the quality of the studies submitted^14^.

The approval of the preliminary research project by a Research Ethics Committees has been mandatory in Brazil^3^^,^ 8. However, some journals did not require that research projects should be approved by a committee. The values found in this study are higher than Sanderberg et al (2002)^10^, showing progress. The Research Ethics Committees act as ghost authors^12^, because when ethical corrections are made, methodological misconceptions are disclosed and suggests corrections that may influence positively the research.

In relationship to the documents related to research ethics in Brazil, the Declaration of Helsinki, in both groups, showed low observance in submitted articles, similar to those found in a paper in 1999^11^ that, after 16 years, there were no significant changes regarding the importance assigned to this declaration. In relation to Resolution 466/12 - the most preponderant Brazilian document of ethics in research - only one journal quoted it, showing that after two years of its publication little was assimilated by Brazilian surgical journals, and this can be proven by the fact that almost 40% of journals still use the Resolution 196/96 in their instructions.

The informed consent is a document with irrefutable ethical value and, usually, the only connection between the research subject and the research itself^8^. Based on this document, the research subject acknowledges the research in which he/she is willing to participate, his/her rights and duties, in addition to the safety provided by this instrument, which ensures the person the possibility of refusing to participate in the research at any time^6^. Despite its importance, this was one of the criterion that has obtained the lowest score in both groups.

This fact may be due to the journals' assumption that the approval of an article by an committee depends on the approval of the informed consent model used by such committee, rendering this information unnecessary^14^; however, it is known that changes can occur in the middle of research and the previous approved protocol may differ from implemented.

Conflicts of interest are a major ethical point, as the manipulation of results by the pharmaceutical industry and major biotechnology companies may compromise thousands of people^2^. Information about possible connections between the researchers and these institutions should be detailed in the articles and is of great relevance to readers, in order to filter the results of the research. According to the results of this research, all the journals requested clarification of potential conflicts of interest; previous researches^10^^,^ 11 ^,^ 14, with similar methodology, had lower results, showing great progress in this regard.

The registration of clinical trials is a new system in Brazil, started in 2007^14^. Albeit recent, this registry is important and of great value, as it prevents the same research being performed more than once, since information on registered clinical trials is available to the public on the website. This registry serves to inform research subjects about the status of the research in which they are participating, as well as to disclose and benefit the most from the results of these studies. However, even at short existence, only three journals not met this requirement.

The two groups showed no significant differences, demonstrating that in national surgery journals there is a certain pattern in the editorial policy regarding ethics of the instructions; however, some aspects need to be improved, as greater use of informed consent and updating instructions request to be followed resolution 466/12.

It must also be emphasized that this research was limited to the study of the ethical requirements present in the instructions to authors, and the editorial reality may differ from that described in these documents^14^. These ethical requirements, due to their high scientific relevance, should be provided in the instructions to authors, from which researchers base conduct their project and research.

## CONCLUSION

There was respect for the most ethical questions evaluated, with no difference between the journals of general or specialties nature. However, it´s necessary to awareness of the editors for continuous updating of the Instructions to Authors and search for possible gaps to minimize possible bioethical abuses of research subjects.
